# Supplementary material for: ATAD3A oligomerization promotes neuropathology and cognitive deficits in Alzheimer’s disease models
Source: Nat Commun. 2022 Mar 2;13:1121. doi: 10.1038/s41467-022-28769-9 (PMC8891325; doi:10.1038/s41467-022-28769-9)
Supplement: Supplementary file 1 — Supplementary information [file 41467_2022_28769_MOESM1_ESM.pdf]

**ATAD3A oligomerization promotes neuropathology and cognitive deficits in Alzheimer's disease models**

Yuanyuan Zhao[1,#], Di Hu[1,#], Rihua Wang[1,#], Xiaoyan Sun[1], Philip Ropelewski[1], Zita Hubler[2], Kathleen Lundberg[3], Quanqiu Wang[4], Drew J. Adams[2], Rong Xu[4], and Xin Qi[1,*]

[1]Department of Physiology & Biophysics; [2]Department of Genetics; [3]Proteomic center; [4]Center for Artificial Intelligence in Drug Discovery, Case Western Reserve University School of Medicine, Cleveland, OH 44106, USA

***Corresponding author:**
Xin Qi Ph.D., Department of Physiology and Biophysics, Case Western Reserve University School of Medicine, 10900 Euclid Ave, E516, Cleveland, Ohio, 44106-4970, USA. Tel: 216-368-4459; Fax: 216-368-5586; E-mail: xxq38@case.edu

#, These authors contributed equally.

**Supplementary Table 1: Primers used for qPCR**

| Gene name | Forward primer | Reverse primer |
|---|---|---|
| Srebf2 | ATGATCACCCCGACGTTCAG | GGTCGCTGCGTTCTGGTATATC |
| Hmgcr | TTGGTCCTTGTTCACGCTCAT | TTCGTCCAGACCCAAGGAAAC |
| Ldlr | ACCTGCCGACCTGATGAATTC | GCAGTCATGTTCACGGTCACA |
| Lxr-alpha | AGCGTCCATTCAGAGCAAGTG | CACTCGTGGACATCCCAGATCT |
| Lxr-beta | ACTCGGAGCAGGTCTTTGCAT | CCTACTCGTGCACATCCCAGAT |
| Apoe | GGCCCAGGAGAATCAATGAG | CCTGGCTGGATATGGATGTTG |
| Abca1 | AGGCCGCACCATTATTTTGTC | GGCAATTCTGTCCCCAAGGAT |
| Lrp1 | ACTATGGATGCCCCTAAAACTTG | GCAATCTCTTTCACCGTCACA |
| Abcg1 | GTGGATGAGGTTGAGACAGACC | CCTCGGGTACAGAGTAGGAAAG |
| Apoa2 | CTGACCTGACAAGGGGTGTC | ATGGCAAAGATTTGGTGGAG |
| Lipe | CCTGTCTCGTTGCGTTTGTA | ACGCTACACAAAGGCTGCTT |
| Snx17 | CAGGGGTCAAAGAGAACAGC | GTGAATGGAGTCCTGCACTG |
| Prkag2 | GGTGTTGACGGAGAAGAGGA | TCATCCAAAGAGTCTTCGCC |
| Prkaa1 | GTCAAAGCCGACCCAATGATA | CGTACACGCAAATAATAGGGGTT |
| Ubc-7 | CTGGCAGAACTCAACAAAAATCC | AGATGAGCCTTAAAAACACCACC |
| Gp78 | ACAAAGACCTATCTGAAACGTCC | AGGGAGCTTGTGGCTCAGTA |
| Gapdh | GACTTCAACAGCAACTCCCAC | TCCACCACCCTGTTGCTGTA |
| Cyp46a1 | TCCTCTCCTGTTCAGCACC | CAGCTTGGCCATGACAACT |
| Cyp51a1 | CTGCCCGCTGGAGCGAAAAG | CACAGGTGTTGTCAGCCGACC |
| Hmgcs1 | GGAAGCCTTTGGGGACGTTA | ACACTCCAACCCTCTTCCCT |

## Supplementary Fig. 1

**a**

Numbers of sub-network nodes and edges

| Subnetwork | Nodes | Nodes | Edges |
|---|---|---|---|
| Chemical-gene network | 473,602 chemicals | 18,701 genes | 15,473,939 |
| Mouse Phenotype-gene network | 9,982 phenotypes | 11,021 genes | 517,381 |
| Gene-gene interaction network | 22,982 genes | 22,982 genes | 382,256 |
| Gene-pathway network | 8,868 genes | 1329 pathways | 66,293 |

**b**

Decile ranking of 13 AD-associated phenotypes among a total of 10,072 prioritized phenotypes

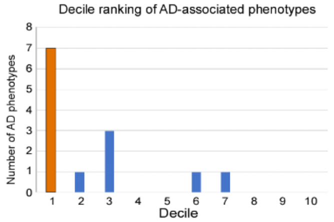


| AD Phenotype | Rank (top%) | AD Phenotype | Rank (top%) |
|---|---|---|---|
| abnormal synaptic transmission | 3.19 | abnormal long term depression | 12.46 |
| neurodegeneration | 3.53 | amyloidosis | 24.30 |
| gliosis | 3.97 | amyloid beta deposits | 28.18 |
| neuron degeneration | 4.16 | tau protein deposits | 28.66 |
| astrocytosis | 5.81 | neurofibrillary tangles | 53.65 |
| abnormal long term potentiation | 8.97 | neurofibrillary tangles | 53.65 |
| microgliosis | 9.71 | cerebral amyloid angiopathy | 68.65 |

**c**

Decile ranking of 22 AD-associated genes among a total of 23,499 prioritized genes

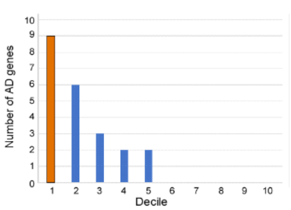


| AD Gene | Rank (top%) | AD Gene | Rank (top%) |
|---|---|---|---|
| VEGFA | 0.06 | HFE | 11.83 |
| NOS3 | 0.09 | TF | 12.17 |
| TNF | 0.13 | ADAM10 | 15.56 |
| PLAU | 0.51 | MPO | 16.89 |
| ACE | 0.62 | BLMH | 21.69 |
| PRNP | 0.67 | MT-ND1 | 21.97 |
| APP | 0.84 | PLD3 | 28.21 |
| PSEN1 | 4.68 | A2M | 33.80 |
| APOE | 6.57 | APBB2 | 35.16 |
| PSEN2 | 10.55 | ABCA7 | 41.55 |
| PAXIP1 | 10.82 | SORL1 | 44.48 |

**d**

Top 20 ranked pathway for input ATAD3A among 1,329 prioritized pathways

| Rank | Pathway |
|---|---|
| 1 | **Protein metabolism** |
| 2 | Diabetes pathways |
| 3 | Unfolded Protein Response |
| 4 | Immune System |
| 5 | C-MYB transcription factor network |
| 6 | Adaptive Immune System |
| 7 | Ubiquitination & Proteasome degradation |
| 8 | HDAC Class I |
| 9 | Generic Transcription Pathway |
| 10 | Huntington's disease |
| 11 | Parkinson's disease |
| 12 | CARM1 and Regulation of the Estrogen Receptor |
| 13 | nuclear estrogen receptor alpha network |
| 14 | Oxidative phosphorylation |
| 15 | Axon guidance |
| 16 | **Alzheimer's disease** |
| 17 | Membrane Trafficking |
| 18 | Respiratory electron transport, ATP synthesis |
| 19 | TGF-beta signaling pathway |
| 20 | Mitochondrial protein import |

**Supplementary Figure 1: Computational analysis was performed to determine the relationship between ATAD3A and AD.** (a) The integrated gene-pathway-phenotype network with labeled data resources was used to analyze the relationship between ATAD3A and AD. (b) AD-specific phenotypes ranked highly for ATAD3A. Seven out of the 13 AD-associated phenotypes ranked within the top 10% (first decile) of a total of 10,072 prioritized phenotypes. For example, phenotypes "abnormal synaptic transmission" and "neurodegeneration" ranked in the top 3.19% and 3.53%, respectively. On average, AD-specific phenotypes ranked in the top 20.82%, which was significantly higher than random ranking ($p = 2.02E-4$). These results indicated that ATAD3A is related to AD at a phenotypic level. (c) AD-specific genes ranked highly for ATAD3A. Nine out of the 22 AD-associated genes ranked within the top 10% (first decile) of a total of 23,499 prioritized genes. For example, genes "VEGFA," "NOS3," and "APP" ranked in the top 0.06%, 0.09%, and 0.84%, respectively. On average, AD genes ranked in the top 14.49%, which was significantly higher than random ranking ($p = 1.34E-10$). These results indicated that ATAD3A is related to AD at a genetic level. (d) The top 20 ranked pathways for the input ATAD3A are shown. These pathways were associated with protein metabolism, immune response regulation, and neurodegeneration.

# Supplementary Fig. 2

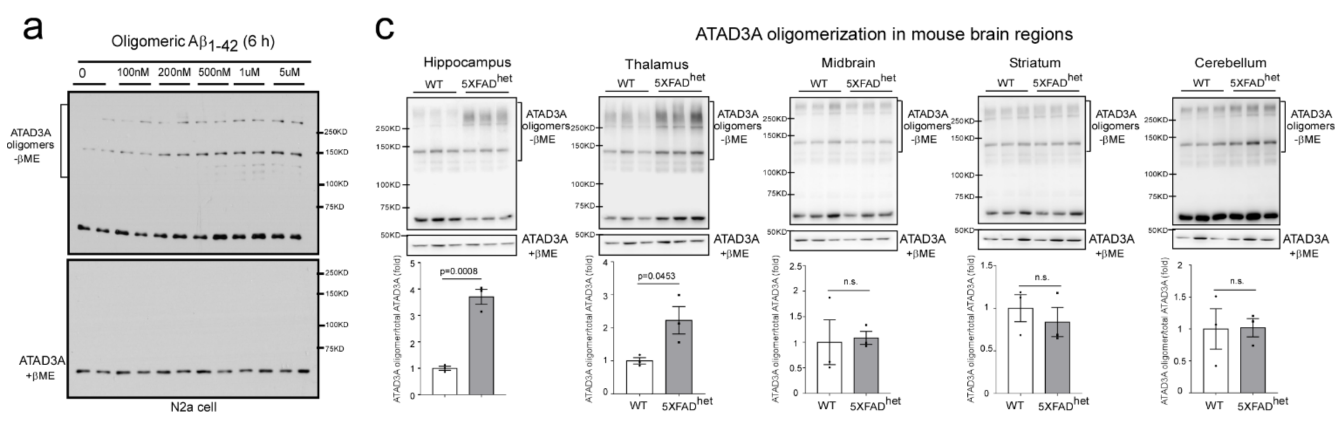


b Information of postmortem brain samples of AD patients and normal subjects

| Type of Biospecimens | Diagnosis | Neurpath |
|---|---|---|
| hippocampus | normal | |
| hippocampus | normal | |
| hippocampus | normal | |
| hippocampus | normal | |
| hippocampus | normal | |
| hippocampus | normal | |
| hippocampus | AD, stroke, dementia | |
| hippocampus | AD | early onset,Braak stage 6 |
| hippocampus | AD | AD, FTD,Braak stage 7 |
| hippocampus | AD | AD, stroke, 5-6 stage |
| hippocampus | AD (B3) | Braak stage 3 |
| hippocampus | AD (B1, C1) | Braak statege 1 |
| hippocampus | AD | |
| hippocampus | AD | AD, 4-5 stage |
| hippocampus | AD | early onset, Braak stage 5 |
| hippocampus | AD (A2, B3,C3) | Braak stage 3 |
| hippocampus | AD (A3, B3, C3) | Braak stage 3 |
| hippocampus | AD (A3, B3,C3) | Braak stage 3 |
| Parietal Cortex | normal | |
| Parietal Cortex | normal | |
| Parietal Cortex | normal | |
| Parietal Cortex | normal | |
| Parietal Cortex | Alzheimer's Disease | |
| Parietal Cortex | Alzheimer's Disease | |
| Parietal Cortex | Alzheimer's Disease | |
| Parietal Cortex | Alzheimer's Disease | |
| Parietal Cortex | Alzheimer's Disease | |

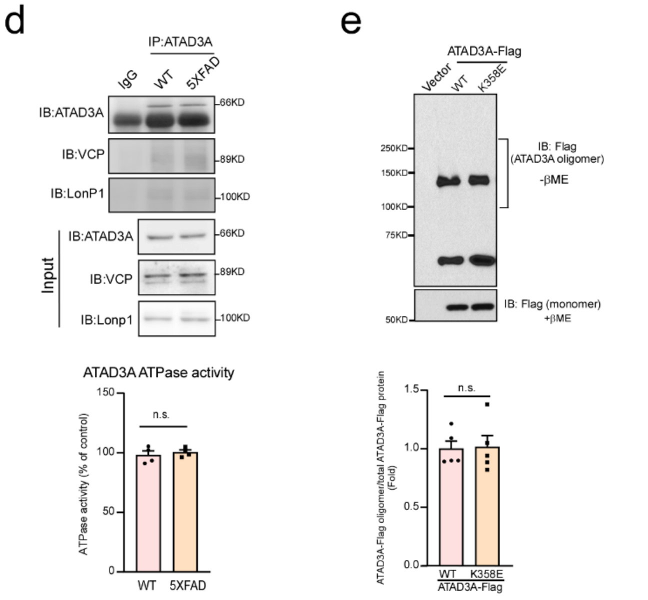


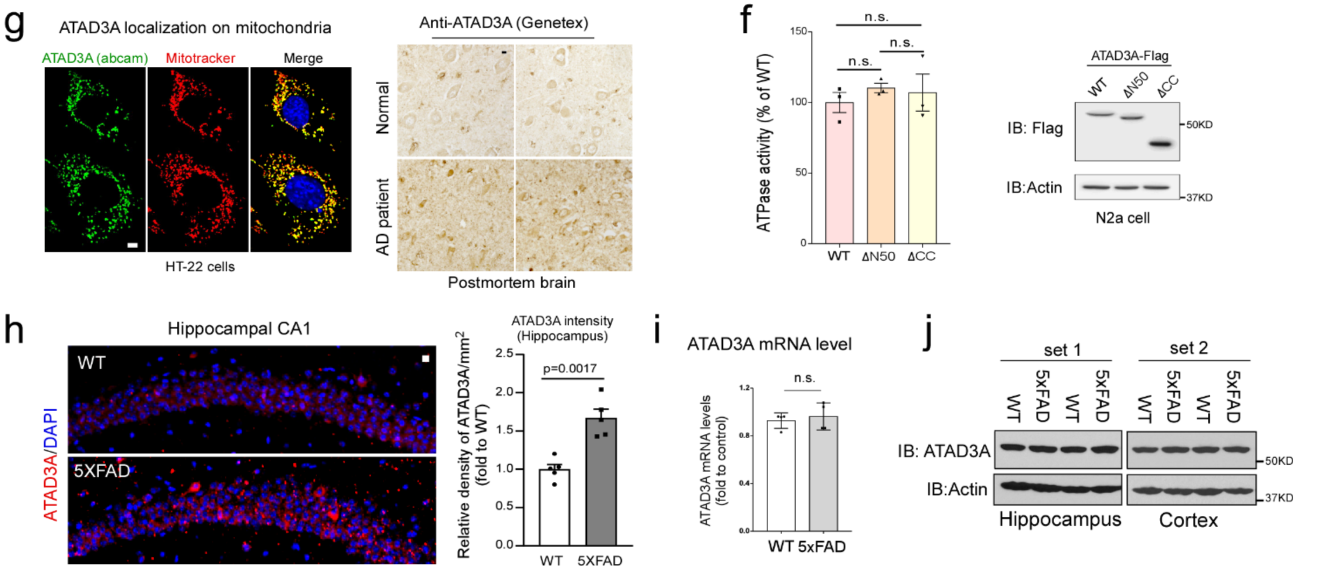

**Supplementary Figure 2: Aberrant ATAD3A oligomerization is associated with AD.** (a) Neuro2a (N2a) cells were treated with oligomeric A$\beta_{1\text{–}42}$ peptides at the indicated dosages. ATAD3A protein levels were determined by western blotting (WB) with anti-ATAD3A antibody in the presence or absence of β-Mercaptoethanol (β-ME). (b) Information for the postmortem brain samples of AD patients and normal subjects. (c) Protein lysates were harvested from the hippocampus, thalamus, midbrain, striatum and cerebellum of three-month-old WT and 5XFAD mice. ATAD3A oligomers were determined by WB with anti-ATAD3A antibody in the absence of β-ME. The histograms show the relative density of the ATAD3A oligomers relative to total ATAD3A levels in the presence of β-ME (n=3 mice/group). (d) Immunoprecipitation of ATAD3A in six-month-old WT and 5XFAD mouse cortex with anti-ATAD3A antibodies, followed by assessing ATPase activity with the immunoprecipitants, was carried out. n=3 mice/group. (e) HEK293T cells were transfected with indicated plasmids for 48h. Western blot was performed with anti-Flag antibody in the presence or absence of β-ME. n=5 independent biological experiments. (f) Left: Immunoprecipitation of ATAD3A in Neuo2a cells expressing ATAD3A-WT-Flag, ATAD3A-ΔN50-Flag or ATAD3A-ΔCC-Flag was carried out. ATPase activity was measured with the immunoprecipitants. n=3 independent experiments. (g) Left: HT-22 cells were stained with ATAD3A (ab112572, Abcam) and Mitotracker Red to confirm that the antibody for ATAD3A staining functions well. Right: Human postmortem brain sections were stained with anti-ATAD3A antibody (GTX116301, GeneTex). (h) Brain sections from three-month-old WT and 5XFAD mice were stained with anti-ATAD3A antibodies. The ATAD3A immunodensity in the CA1 region of the hippocampus was quantified (n = 5 mice/group). Scale bar: 10 µm. (i) mRNA level of ATAD3A was measured in the cortex of WT and 5XFAD mice (n=3 mice/group). (j) Total protein lysates were harvested from the cortex and hippocampus of three-month-old WT and 5XFAD mice (n = 4 mice/group). WB was performed with the indicated antibodies. Representative blots from at least three independent experiments are shown. The data in panels c, d, e, h, i were compared by the unpaired Student's *t*-test (two-tailed), and the data in panel f were compared by one-way ANOVA with Dunnett's multiple comparison's test.

# Supplementary Fig. 3

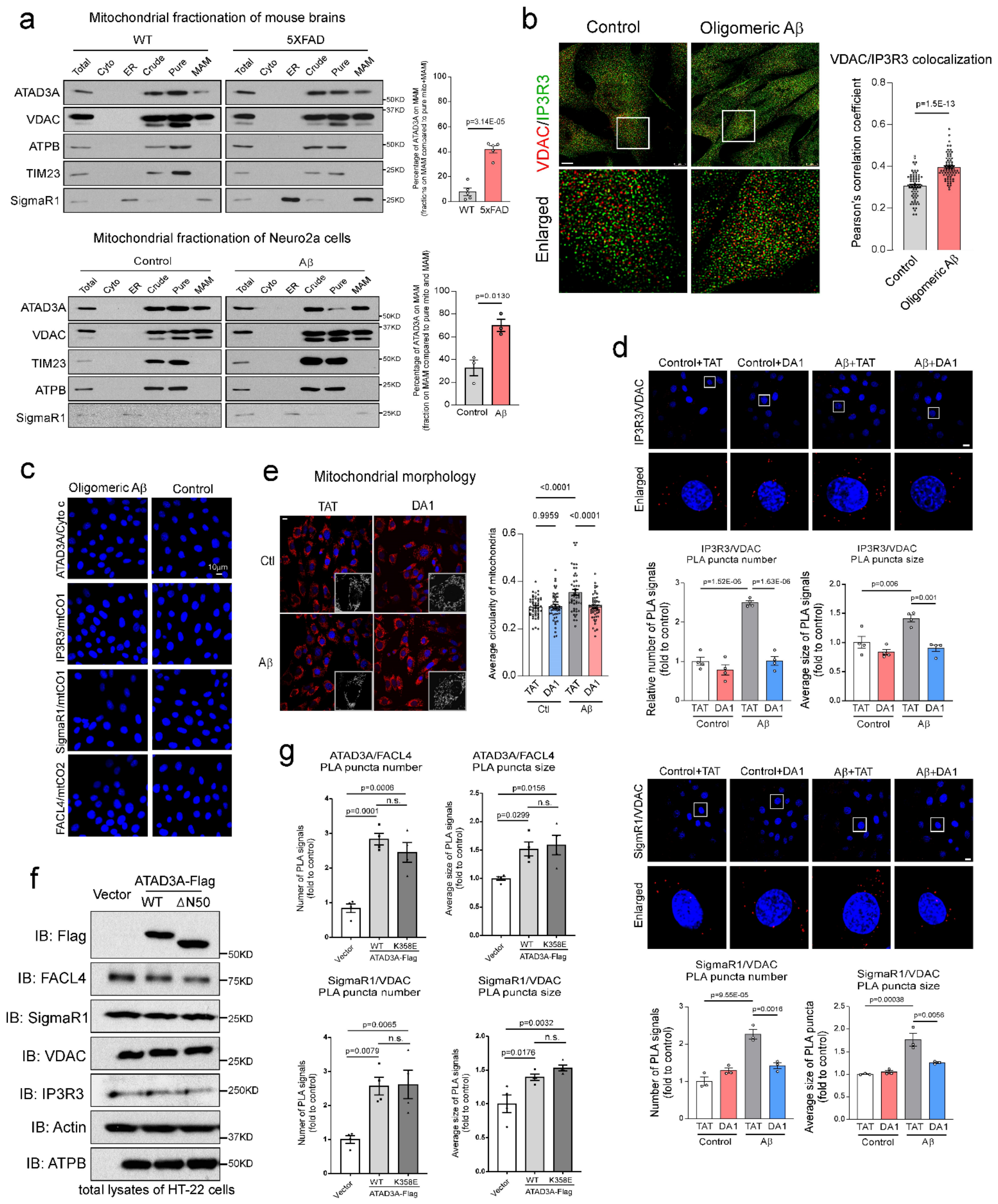

**Supplementary Figure 3: ATAD3A is present at the MAMs.** (a) Mitochondrial sub-compartmental fractions of three-month-old WT and 5XFAD mouse brain (upper panel) (n=5) and oligomeric Aβ-treated Neuro2a cells (lower panel) (n=3) were prepared. WB analysis was performed with the indicated antibodies (mouse brain: n=5; cell culture: n=3 independent biological experiments). Crude and pure labelled on the images indicates crude and pure mitochondria, respectively. (b) HT-22 cells were treated with oligomeric $A\beta_{1-42}$ peptides (5 μM for 18 h). Cells were then stained with the indicated antibodies. The co-localization of IP3R3 and VDAC was imaged by high-resolution confocal microscopy and assessed by the Pearson's correlation coefficient. At least 50 cells/group were analyzed. Scale bar: 5 μm. (c) Cells were stained with the indicated antibodies and then subjected to PLA analysis. (d) HT-22 cells were treated with peptide DA1 or control peptide TAT (1 μM, each) followed by the addition of oligomeric $A\beta_{1-42}$ peptides (5 μM) for 18 h, and were stained with indicated antibodies and subjected to PLA analysis. Histogram: quantification of the number and size of PLA-positive puncta (red). n=4 for IP3R3/VDAC, n=3 for SigmaR1/VDAC. At least 200 cells/group were analyzed. Scale bar: 10 μm. (e) Cells were stained with anti-Tom20 antibody. Fragmented mitochondria were quantified. At least 50 cells/group was counted. Scale bar: 10 μm. (f) HT-22 cells were transfected with the indicated plasmids for 48 h. WB was performed with the indicated antibodies. Representative images and blots from at least three independent experiments are shown. (g) HT-22 cells were transfected with the indicated plasmids for 48 h. Cells were stained with the indicated antibodies and subjected to PLA analysis. Histogram: quantification of the number and size of PLA-positive puncta (red). n=4 for each group. At least 200 cells/group were analyzed. The data are presented as the mean ± SEM. Representative images and blots from at least three independent experiments are shown. The data in panels a and b were compared by the unpaired Student's *t*-test (two-tailed), and the data in panels d, e, g were compared by one-way ANOVA with Tukey's multiple comparisons test.

# Supplementary Fig. 4

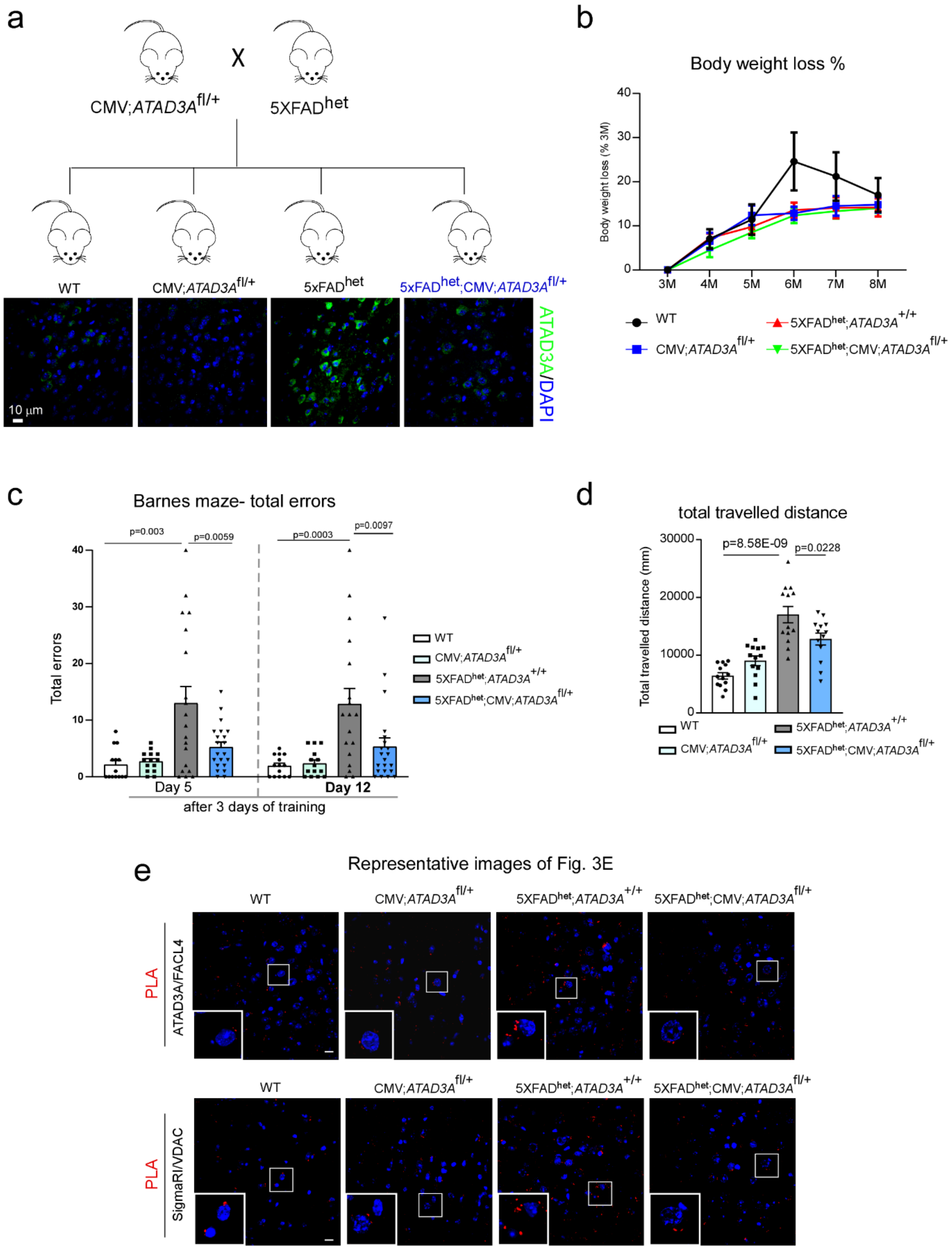

**Supplementary Figure 4: The effects of ATAD3A heterozygous knockout in 5XFAD mice.** (a) Upper panel: Schematic diagram of the generation of ATAD3A heterozygous knockout 5XFAD mice. Bottom panel: brain sections from three-month-old mice of the indicated genotypes were stained with anti-ATAD3A antibodies (green). Scale bar: 10 µm. (b) Body weights of mice of the indicated genotypes were recorded with age. n=15 mice for WT, n=18 mice for CMV;$ATAD3A^{fl/+}$, n=24 mice for $5XFAD^{het}$;$ATAD3A^{+/+}$ and n=21 mice for $5XFAD^{het}$;CMV;$ATAD3A^{fl/+}$ . (c) The Barnes maze test was administered to eight-month-old mice of the indicated genotypes (n=14 mice for WT group, n=16 mice for CMV;$ATAD3A^{fl/+}$group, n=19/18 mice for $5XFAD^{het}$;$ATAD3A^{+/+}$in day 5/day 12 test and n=21 mice for $5XFAD^{het}$;CMV;$ATAD3A^{fl/+}$ group). (d) The open-field test was administered to six-month-old mice at the indicated genotypes. The total traveled distances are shown (n = 13 mice/group). (e) Representative images for Fig. 3e. Scale bar: 10 µm. All the data are presented as the mean ± SEM and compared by two-way ANOVA (b-c) or one-way ANOVA (d) with Tukey's multiple comparisons test.

Supplementary Fig. 5

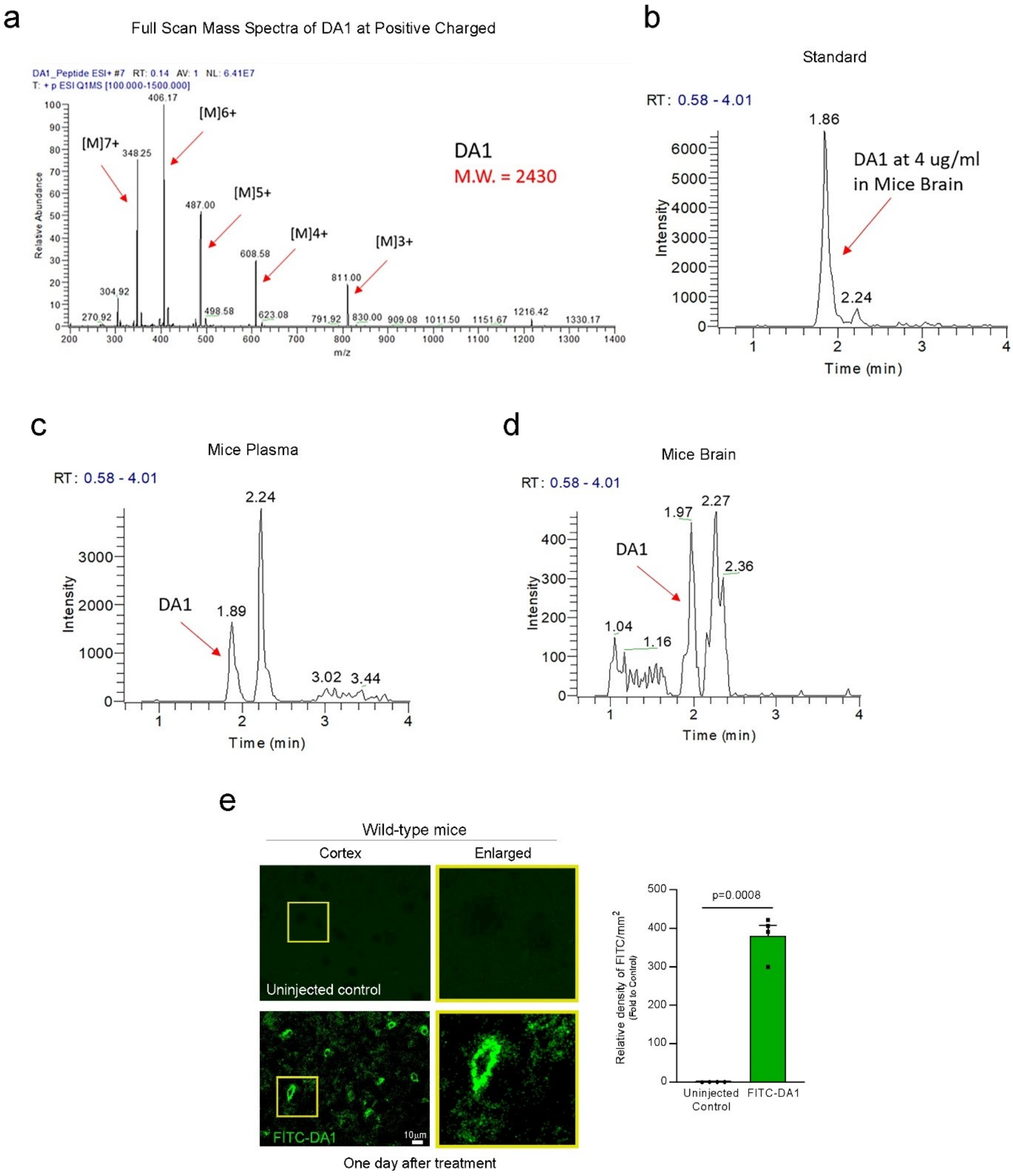

**Supplementary Figure 5: DA1 enters the brains of AD mice.** LC/MS/MS Chromatograms of DA1 in mouse Samples. (a) DA1 at six charged in positive mode was analyzed on a triple quadrupole mass spectrometer (TSQ Quantiva, Thermos Fisher Scientific) using Selective Reaction Monitoring (SRM) with the transition at m/z 406.2 > 257.5. The LC/MS/MS chromatograms of DA1 in samples are shown for (b) DA1 added to the brain homogenate, (c) DA1 detected in plasma and (d) DA1 detected in brain homogenate after intravenous administration of DA1 (10 mg/kg) in mice. (e) WT mice were subcutaneously treated with FITC-conjugated DA1 (1 mg/kg/day) using an osmotic minipump. Mouse brain sections were imaged by microscopy. The FITC fluorescence density was quantified from three separate fields of mouse cortex (n=4, the unpaired Student's t-test).

# Supplementary Fig. 6

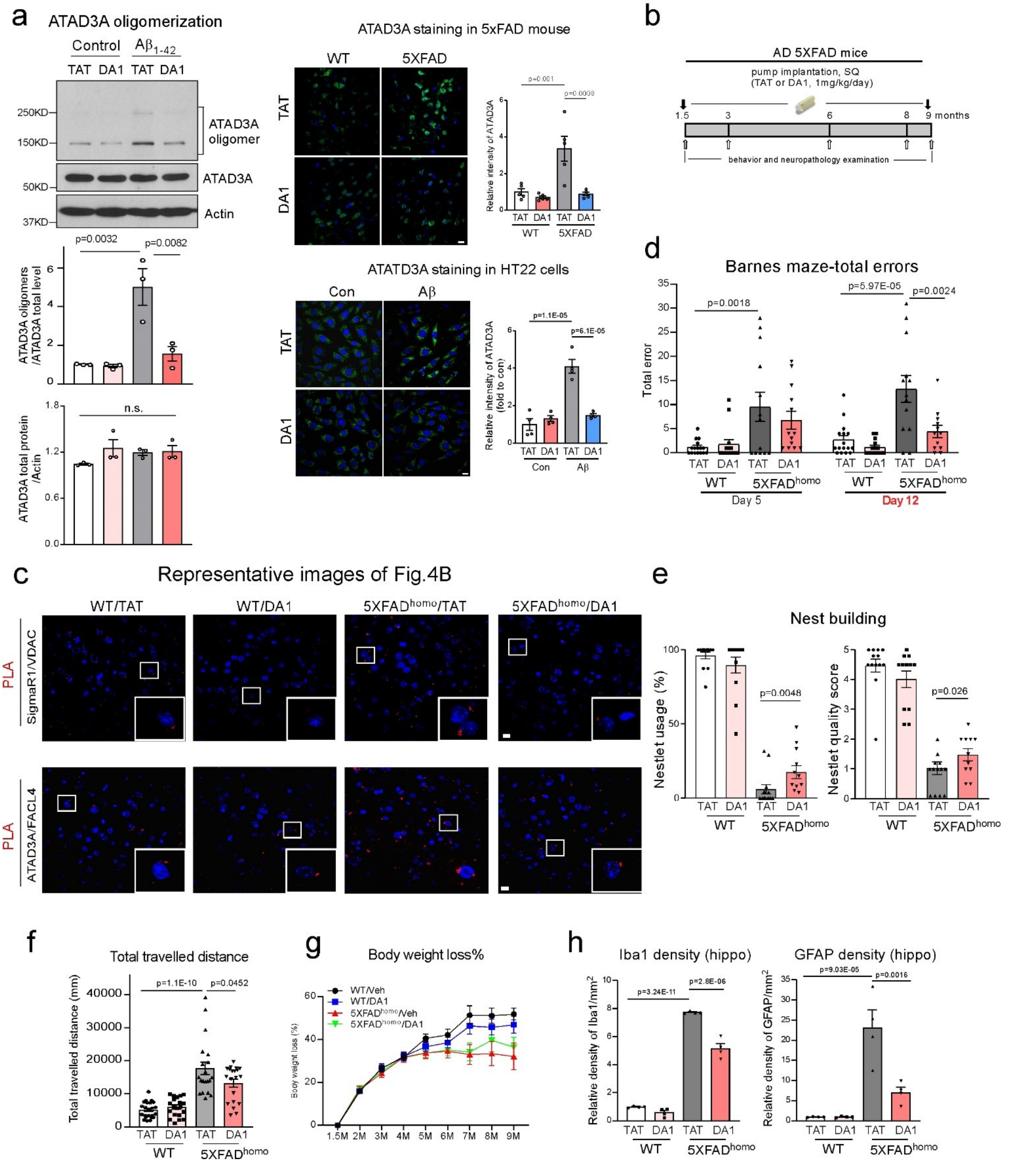

**Supplementary Figure 6: The effects of DA1 treatment on AD cell culture and AD mice.** HT-22 cells were treated with DA1 or TAT peptide (1 μM), followed by treatment with oligomeric A$\beta_{1\text{–}42}$ peptides (Western blot: 5 μM, 9 hours; PLA staining: 5 μM, 18h). (a) Left: ATAD3A oligomerization was assessed by WB under non-reducing conditions. Histogram: ATAD3A oligomers/total ATAD3A levels and ATAD3A total protein/Actin. n=3 independent experiments. Right: brain sections from WT and 5XFAD mice (n=5 mice/group), and HT-22 cells (n=4 independent experiments) were stained with anti-ATAD3A antibody (Abcam). Scale bar: 10 μm. (b) The timeline and administration route of DA1 treatment of 5XFAD and WT mice. (c) Representative images for Fig. 4b. Scale bar: 10 μm. (d) The Barnes maze test was administered to eight-month-old mice (WT/TAT: n=16 mice; WT/DA1: n=14 mice; 5XFAD$^{homo}$/TAT: n = 13 mice; 5XFAD$^{homo}$/DA1: n = 13 mice/day 5 group and n=12 mice/day 12 group). (e) The nest building test was administered to eight and half-month-old mice of the indicated treatment groups (WT/TAT and 5XFAD$^{homo}$/TAT: n = 13 mice/group; WT/DA1: n = 12 mice/group; 5XFAD$^{homo}$/DA1: n=11 mice/group). (f) The open-field test was administered to six-month-old mice of the indicated treatment groups. The total traveled distances are shown (n=23 mice for WT group, and n=20 mice for the other three groups). (g) The body weights of mice of the indicated treatment groups were recorded with age (WT/DA1 group: n = 31 mice, n=34 mice for the other three groups). (h) Brain sections from six-month-old mice of the indicated treatment groups were stained with anti-Iba1 and anti-GFAP antibodies. The relative immunodensities of Iba1 and GFAP in the hippocampus (hippo) were quantified from three separate fields of each mouse (n=4 mice/group). All data are presented as the mean ± SEM and compared by two-way ANOVA (d, g) or one-way ANOVA (a, e-f, h) with Tukey's multiple comparisons test or with correcting for multiple comparisons by controlling the false discovery rate (e).

# Supplementary Fig. 7

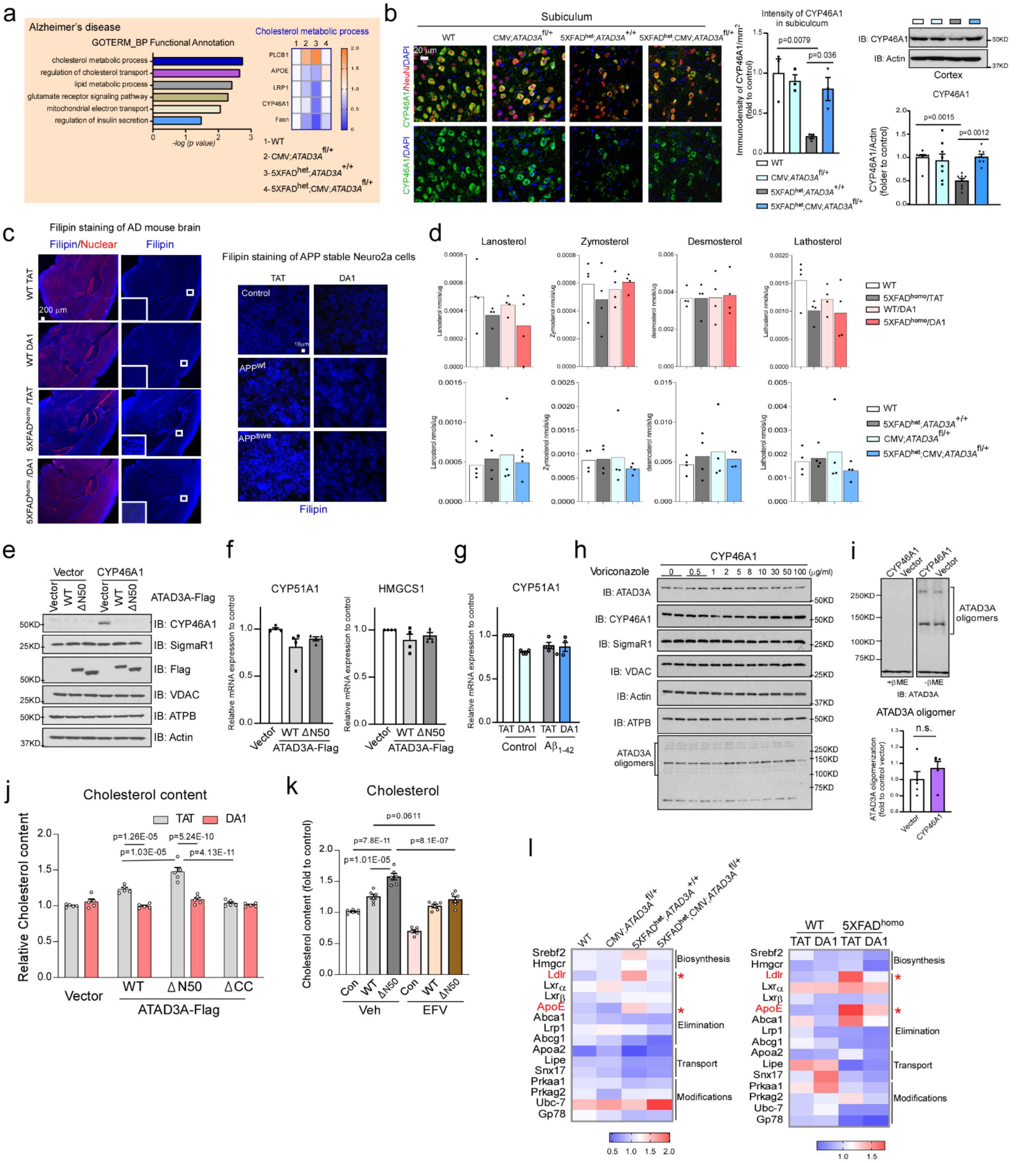

**Supplementary Figure 7: ATAD3A oligomerization impairs brain cholesterol metabolism.** (a) Label-free tandem mass spectrometry of mouse cortex (n = 3 mice/group). Heat map: proteins involved in the "Cholesterol metabolic process". (b) Left: Brain sections from eight-month-old mice were stained with anti-CYP46A1 and anti-NeuN antibodies. The immunodensity of CYP46A1 in $NeuN^+$ cells of mouse subiculum was quantified from two separate fields per mouse (n = 3 mice/group). Scale bar: 20 µm. Right: western-blot (WB) was performed. n=8 mice/group. (c) Left: brain sections from nine-month-old mice were stained with the filipin probe (blue). Right: stable APP Neuro2a cells were treated with DA1 or TAT (1 µM for 3 days) and stained with filipin probe. Scale bar: 200 µm (left)/10 µm (right). (d) Cortex from six-month-old DA1-treated mice or eight-month-old $5XFAD^{het}$;CMV;$\mathit{ATAD3A}^{fl/+}$ mice was subjected to GC-MS (n = 4 mice/group). Neuro2a cells were transfected with the indicated plasmids for 48 h. (e) WB was performed. (f) qPCR was performed. (g) HT-22 cells were pretreated with DA1 or TAT (1 µM) followed by $A\beta_{1\text{–}42}$ treatment (5 µM) for 9 h. qPCR was performed. n=4 independent biological experiments (f-g). (h) HT-22 cells were infected with CYP46A1 lentivirus followed by voriconazole treatment for 24 h. WB was performed. (i) ATAD3A oligomerization was assessed by WB. n=5 independent biological experiments. (j) Neuro2a cells were transfected with the indicated plasmids for 24 h followed by treatment with DA1 or TAT (1 µM for 2 days). Cholesterol content was measured (n=5). (k) Neuro2a cells were transfected with the indicated plasmids for 24 h followed by treatment with EFV (5 µM for 2 days). Cholesterol content was measured (n=6). Con: control vector. (l) Genes involved in cholesterol metabolism were analyzed by qPCR (n=3 mice/group). Heat map: the mean of the genes analyzed. *, $p < 0.01$ ($5XFAD^{het}$;$\mathit{ATAD3A}^{+/+}$ vs. $5XFAD^{het}$;CMV;$\mathit{ATAD3A}^{fl/+}$ mice, or TAT- vs. DA1-treated 5XFAD mice). Representative images and blots from at least three independent experiments are shown. Data are the mean ± SEM, and were compared by unpaired Student's t-test (two-tailed, panel i) and one-way ANOVA with Tukey's multiple comparisons test (the rest of panels).

# Supplementary Fig. 8

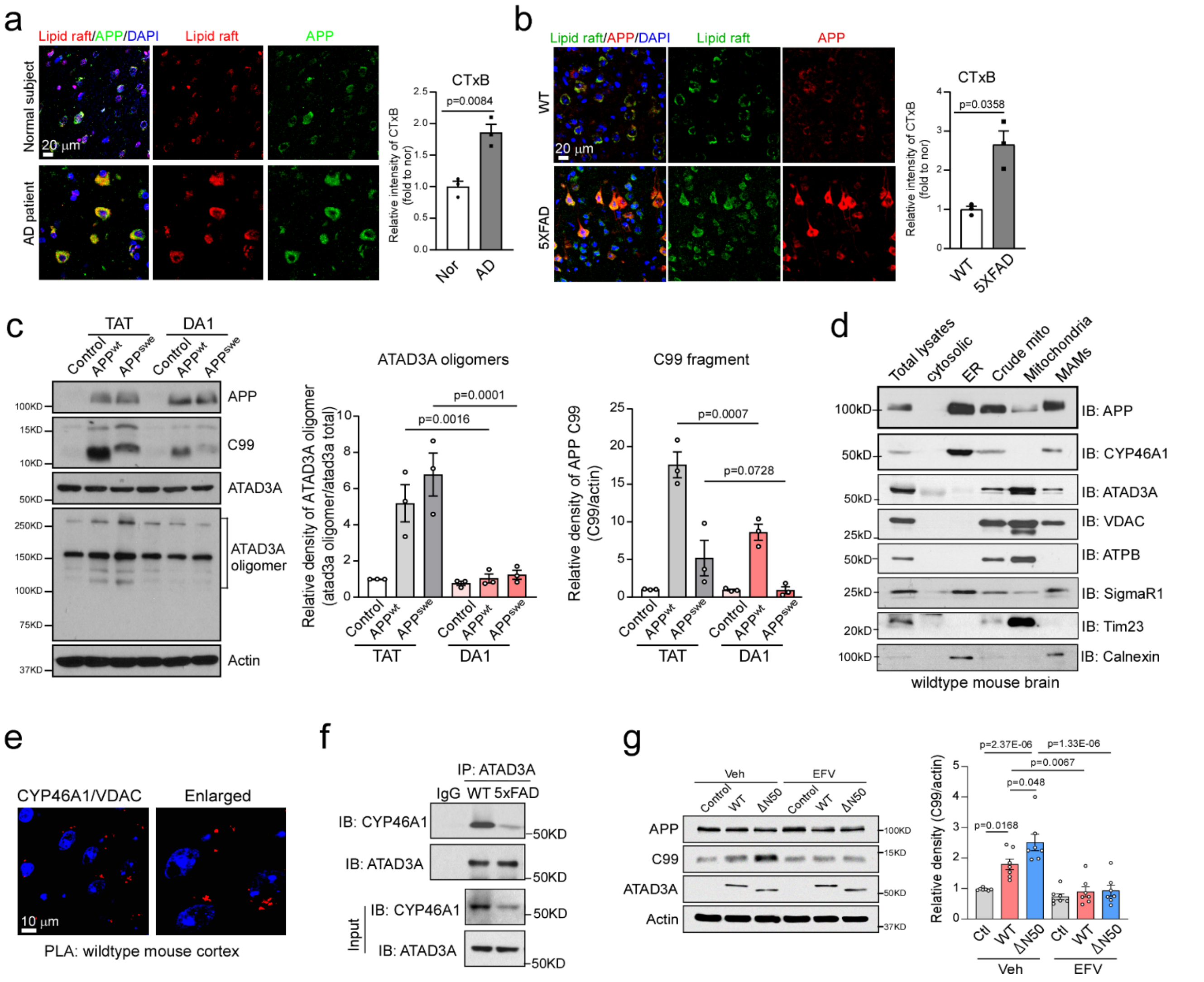


**Supplementary Figure 8: ATAD3A oligomerization mediates APP processing.** (a) Postmortem cortex sections from normal subjects (Nor) and AD patients and (b) brain sections from three-month-old WT and 5XFAD mice were stained with anti-CTxB and anti-APP antibodies. CTxB immunodensity was quantified from three separate fields of each patient (n=3)/mice (n=3). (c) Stable $APP^{wt}$- and $APP^{swe}$-expressing Neuro2a cells were treated with DA1 or TAT (1 μM/day for 3 days). WB was performed. Histograms: the relative densities of the ATAD3A oligomers and C99 fragment (n=3 independent experiments). (d) WB analysis of mouse brain mitochondrial sub-compartmental fractions using the indicated antibodies. (e) Brain sections from three-month-old WT mice were stained with anti-CYP46A1 and anti-VDAC antibodies and then subjected to PLA analysis. (f) Total protein lysates were harvested from the cortex of six-month-old WT and 5XFAD mice. Immunoprecipitation was performed with an anti-ATAD3A antibody, followed by WB. (g) Stable $APP^{wt}$-expressing cells were transfected with the indicated plasmids, followed by the treatment with Efavirenz (EFV) (5 μM for 2 days). Control and Ctl on the image indicate control vector. The product of APP processing, C99, was assessed by WB. Histogram: the relative density of C99 to actin (n=7 independent experiments). Representative images and blots from at least three independent experiments are shown. All data are presented as the mean ± SEM. Data in panels a and b were compared by unpaired student t-test (two-tailed), and data in panels c and g were compared by one-way ANOVA with Tukey's multiple comparisons test.

# Supplementary Fig. 9

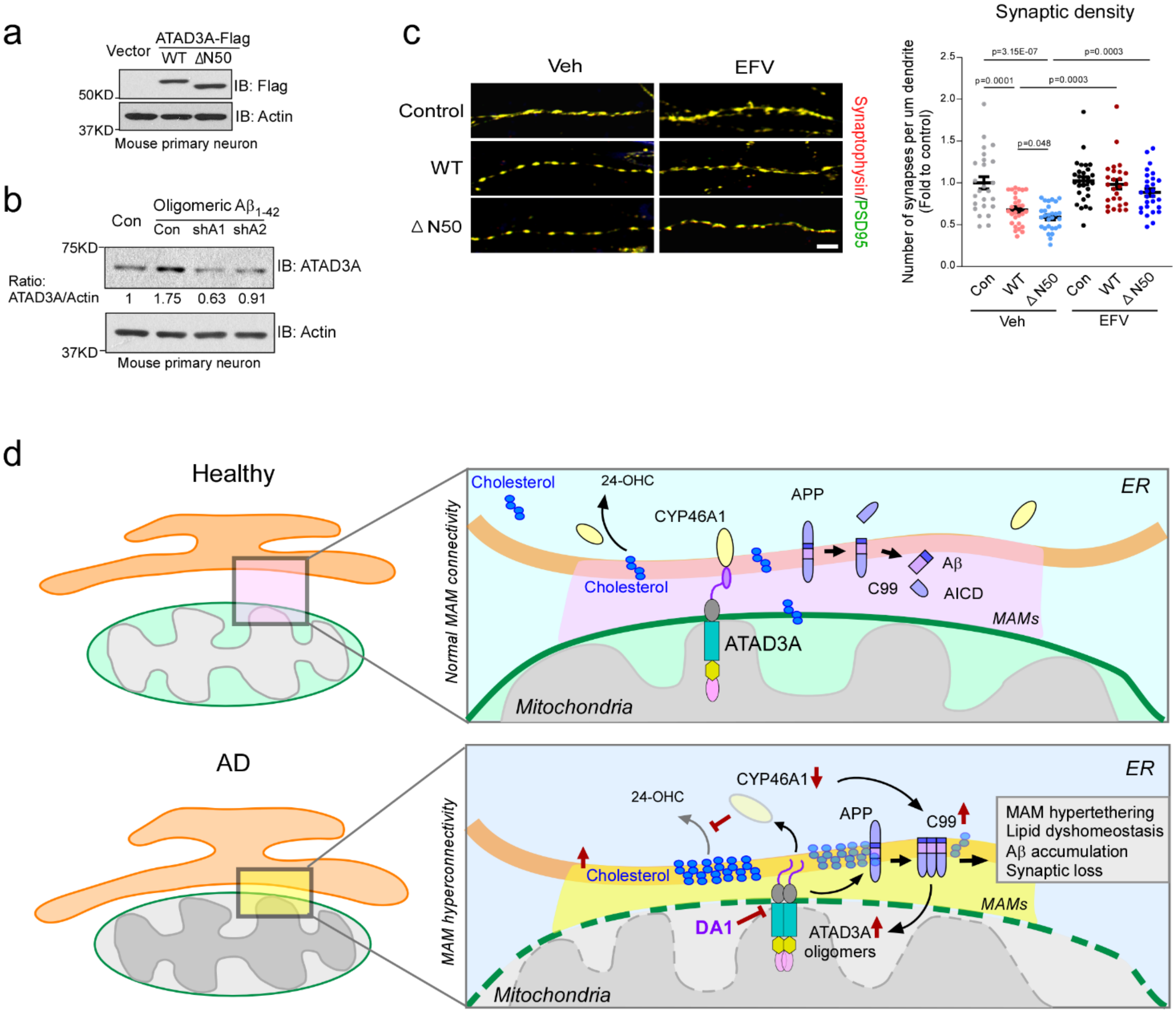


**Supplementary Figure 9: Effects of ATAD3A oligomerization on synaptic integrity of primary mouse cortical neurons.** (a) Primary mouse cortical neurons were expressed with ATAD3A-WT-Flag, ATAD3A-ΔN50-Flag, or control vector for 48 h. Total cell lysates were harvested, and WB was performed to confirm the expression of Flag-tagged ATAD3A. (b) Primary mouse cortical neurons were infected with control (Con) or ATAD3A shRNA (shA1 and shA2) lentivirus for 48 h. Total cell lysates were harvested, and WB was performed to confirm ATAD3A knockdown. Representative images and blots from at least three independent experiments are shown. (c) Primary mouse cortical neurons were expressed with ATAD3A-WT-Flag (WT), ATAD3A-ΔN50-Flag (ΔN50), or control vector (Con) for 24 h. The cells were then treated with Efavirenz (EFV) (5 μM for 2 days). The synaptophysin$^{+}$PSD95$^{+}$ clusters along the dendrites were counted, and the number of synapses per micron of dendrites was quantified. n=25-29 neurons were collected from at least three independent experiments. Scale bar: 5 μm. All data are presented as the mean ± SEM and compared by one-way ANOVA with Tukey's multiple comparisons test. (d) The scheme of the study.
