# Supplementary material for: ATAD3A oligomerization promotes neuropathology and cognitive deficits in Alzheimer’s disease models
Source: Nat Commun. 2022 Mar 2;13:1121. doi: 10.1038/s41467-022-28769-9 (PMC8891325; doi:10.1038/s41467-022-28769-9)
Supplement: Supplementary file 2 — Reporting Summary [file 41467_2022_28769_MOESM2_ESM.pdf]

nature research

Corresponding author(s): Xin Qi

Last updated by author(s): 2/10/2022

Reporting Summary

Nature Research wishes to improve the reproducibility of the work that we publish. This form provides structure for consistency and transparency in reporting. For further information on Nature Research policies, see our Editorial Policies and the Editorial Policy Checklist.

Statistics

For all statistical analyses, confirm that the following items are present in the figure legend, table legend, main text, or Methods section.

n/a	Confirmed	
☐	☒	The exact sample size (*n*) for each experimental group/condition, given as a discrete number and unit of measurement
☐	☒	A statement on whether measurements were taken from distinct samples or whether the same sample was measured repeatedly
☐	☒	The statistical test(s) used AND whether they are one- or two-sided<br>*Only common tests should be described solely by name; describe more complex techniques in the Methods section.*
☒	☐	A description of all covariates tested
☒	☐	A description of any assumptions or corrections, such as tests of normality and adjustment for multiple comparisons
☐	☒	A full description of the statistical parameters including central tendency (e.g. means) or other basic estimates (e.g. regression coefficient) AND variation (e.g. standard deviation) or associated estimates of uncertainty (e.g. confidence intervals)
☐	☒	For null hypothesis testing, the test statistic (e.g. *F*, *t*, *r*) with confidence intervals, effect sizes, degrees of freedom and *P* value noted<br>*Give P values as exact values whenever suitable.*
☒	☐	For Bayesian analysis, information on the choice of priors and Markov chain Monte Carlo settings
☒	☐	For hierarchical and complex designs, identification of the appropriate level for tests and full reporting of outcomes
☒	☐	Estimates of effect sizes (e.g. Cohen's *d*, Pearson's *r*), indicating how they were calculated

Our web collection on statistics for biologists contains articles on many of the points above.

Software and code

Policy information about availability of computer code

Data collection	cSeries Capture Software 1.9.8.0403 (C600 azure biosystem), FV10-ASW 4.2 Viewer (for Fluoview FV1000 confocal microscope imaging), Keyence BZ-X viewer 1.3.1 (Keyence BZ-X710), Enhanced ChemStation E.02.02.1431 (GC/MS).
Data analysis	GraphPad Prism 9.0 (for quantification and statistic analysis), PEAKS 8.5 (for proteomics analysis), Fiji ImageJ 1.5 (for image analysis), Java Runtime Enviroment (JRE) version 8 (for context-sensitive network-based ranking algorithm).

For manuscripts utilizing custom algorithms or software that are central to the research but not yet described in published literature, software must be made available to editors and reviewers. We strongly encourage code deposition in a community repository (e.g. GitHub). See the Nature Research guidelines for submitting code & software for further information.

Data

Policy information about availability of data

All manuscripts must include a data availability statement. This statement should provide the following information, where applicable:

- Accession codes, unique identifiers, or web links for publicly available datasets
- A list of figures that have associated raw data
- A description of any restrictions on data availability

Data supporting the findings of this study are provided within the paper and its supplementary information. Source data are provided with this paper and all statistical data are presented in Source Data file. The mass spectrometry proteomics data have been deposited to the ProteomeXchange Consortium via the PRIDE partner repository with the dataset identifier PXD031523 (http://www.ebi.ac.uk/pride/archive/projects/PXD031523).

Field-specific reporting

Please select the one below that is the best fit for your research. If you are not sure, read the appropriate sections before making your selection.

[x] Life sciences [ ] Behavioural & social sciences [ ] Ecological, evolutionary & environmental sciences

For a reference copy of the document with all sections, see nature.com/documents/nr-reporting-summary-flat.pdf

Life sciences study design

All studies must disclose on these points even when the disclosure is negative.

Sample size	The sample size per group was determined from previous publications with similar methodologies (Zhang et al., 2020, Science Advances; Zhao et al., 2019, Nature communications; Guo et al., 2016, Nature communications; Guo et al., 2013, J. Clin. Invest).<br>For the animal studies, we used n = 13-33 mice/group for the behavioral tests, n = 3–6 mice/group for the biochemical analyses, and n = 3–11 mice/group for the pathology studies. The exact n number was provided in figure legend.
Data exclusions	No samples or animals were excluded from the analysis.
Replication	Replication of experiments was successful in three different experiments/cohort and data is accumulated during the analysis process. Results also were replicated in independent experimental setting. At lease three independent biological replicates were performed for each experiment figure, the n number of independent biological repeats was included in the figure legend.
Randomization	AD mice and their littermate WT mice were collected from each cohort depending on the genotype , randomly allocated and used for the experiment. All WT and AD animals were assigned randomly to TAT or DA1 treatment, and within animal controls were performed wherever possible. All cells analyzed for quantification were randomly selected. All cell and mouse samples were randomly selected for experiments. Human samples were requested and randomly provided by the third-party. Frozen tissue from normal subject and AD patients were randomly diced and homogenated for experiments.
Blinding	For experiments using cell lines, the investigators were not blinded during data acquisition and analysis. The application of treatments and processing procedures negated the possibility of blinding but there was no human bias given all data was collected independently.<br>For all of the animal studies, all animals used were coded. All behavioral analyses were conducted by an experimenter who was blinded to genotypes and treatment groups. Group allocations were decoded afterwards for the purpose of data analysis.<br>For experiments involving human tissues, sample IDs were coded and the investigator was not aware of the group allocation during data acquisition. Group allocations were decoded afterwards for the purpose of data analysis.<br>For all imaging analyses, an observer who was blind to the experimental groups conducted the quantitation.

Reporting for specific materials, systems and methods

We require information from authors about some types of materials, experimental systems and methods used in many studies. Here, indicate whether each material, system or method listed is relevant to your study. If you are not sure if a list item applies to your research, read the appropriate section before selecting a response.

Materials & experimental systems

n/a	Involved in the study	
[ ]	[x]	Antibodies
[ ]	[x]	Eukaryotic cell lines
[x]	[ ]	Palaeontology and archaeology
[ ]	[x]	Animals and other organisms
[ ]	[x]	Human research participants
[x]	[ ]	Clinical data
[x]	[ ]	Dual use research of concern

Methods

n/a	Involved in the study	
[x]	[ ]	ChIP-seq
[x]	[ ]	Flow cytometry
[x]	[ ]	MRI-based neuroimaging

Antibodies

Antibodies used	The antibody against ATAD3A (H00055210-D01, 1:1000) was from Abnova. The antibodies against ATPB (17247-1-AP, 1:1000), SigmaR1 (15168-1-AP, 1:1000), calnexin (10427-2-AP, 1:1000), CYP46A1 (12486-1-AP, 1:1000), and mtCO2 (55070-1-AP, 1:1000) were from ProteinTech. The antibodies against FACL4 (sc-365230, 1:1000), Tim23 (sc-514463, 1:1000), and Tom20 (sc-11415, 1:1000) were purchased from Santa Cruz Biotechnology. The antibodies against APP (ab32136, 1:5000), mtCO1 (ab14705, 1:1000), ATAD3A (ab112572, Abcam), cytochrome C (ab110325, 1:10000), VDAC (ab14734, 1:2000), ClpP (ab124822, 1:2000), and synaptophysin (ab32127, 1:10000) were from Abcam. The antibodies against the C-terminal of APP (A8717, 1:5000), NeuN (A60, MAB377, 1:1000), FLAG (F3165, 1:2000), and β-actin (A1978, 1:10000) were obtained from Sigma-Aldrich. The Iba1 (019–19741, 1:500) antibody was from Wako Chemicals. ATAD3A (GTX116301,1:50) antibody was from GeneTex. The antibodies against GFAP (MAB360, 1:1000) and IP3R3 (AB9076,1:1000) were purchased from Millipore. The CTxB (1:200, bs-12862R) antibody was received

	from Bioss, and the MAP2 antibody (NB300-213, 1:500) was purchased from Novus. The PSD-95 antibody (MA1-045, 1:500) was obtained from Invitrogen. The antibody against purified anti-β-amyloid 1-16 (clone 6E10, #803001, 1:1000) was from BioLegend. The HRP-conjugated anti-mouse (31430, 1:5000) and rabbit (31360, 1:5000) secondary antibodies were from Thermo Fisher Scientific. The VeriBlot secondary antibody (HRP) (ab131366, 1:2000), which does not recognize heavy or light chains, was from Abcam. The Alexa 488 (A11034, 1:1000), 568 (A11031, 1:1000), 405 (A31553, 1:1000) fluorescent secondary antibodies were from Life Technologies and DyLight 405 (703-475-155, 1:500) was from Jackson Immuno Research.
Validation	Anti-ATAD3A (PMID: 30914652), anti-ATPB (PMID: 33398173), anti-SigmaR1 (PMID: 30626719), anti-calnexin (PMID: 30864682), anti-CYP46A1 (PMID: 20039312), anti-mtCO2 (PMID: 30914652), anti-FACL4 (PMID: 33951438), anti-Tim23 (PMID: 33828088), anti-Tom20 (PMID: 34999730), anti-APP (PMID: 33427741), anti-mtCO1 (PMID: 33472069), anti-ATAD3A (PMID: 30914652), anti-cytochrome c (PMID: 33733054), anti-VDAC (PMID: 33164581), anti-ClpP (PMID: 31904544), anti-synaptophysin (PMID: 33473105), anti-APP-C-ter (PMID: 8751438), anti-NeuN (PMID: 26303010), anti-FLAG (PMID: 31427575), anti-β-actin (PMID: 28607478), anti-Iba1 (PMID: 31551601), anti-ATAD3A (PMID:30914652), anti-GFAP (PMID: 26376776), anti-IP3R3 (PMID: 22331418), anti-CTxB (PMID: 28524374), anti-MAP2 (PMID: 34706220), anti-PSD95 (PMID: 31151856), anti-β-amyloid 1-16 (PMID: 26779813), HRP-conjugated anti-mouse (PMID: 30926623), HRP-conjugated anti-rabbit (PMID: 17172443), VeriBlot secondary antibody (HRP) (PMID: 33393215), Alexa 488 (PMID: 28186121), Alexa 568 (PMID: 30531936), Alexa 405 (PMID: 30784582) , DyLight 405 (PMID: 33837051)

Eukaryotic cell lines

Policy information about cell lines

Cell line source(s)	HEK293T cells are from MilliporeSigma (12022001); HT22 cells are from MilliporeSigma (SCC129); Neuro2a cells are from ATCC (CCL-131); Neuro2a cells stably overexpressing human APP wildtype (APPwt) or Swedish mutant (APPswe, K670N and M671L APP, clone Swe.10) were obtained from Dr. Gopal Thinakaran (University of Chicago).
Authentication	All cell lines were authenticated by SNP test. All of the knock-down cell lines were validated by western blot using the antibodies against the protein that were knocked down.
Mycoplasma contamination	All cell lines were tested negative for mycoplasma contamination.
Commonly misidentified lines (See ICLAC register)	No commonly misidentified cell lines were used.

Animals and other organisms

Policy information about studies involving animals; ARRIVE guidelines recommended for reporting animal research

Laboratory animals	All mice were maintained under a 12 h/12 h light/dark cycle (light on at 6 AM and off at 6 PM) with ad libitum access to food and water under the ambient temperature at 23°C and humidity at 40-60%. All animal experimental protocols were approved by the Institutional Animal Care and Use Committee of Case Western Reserve University. Sufficient procedures were employed to reduce the pain and discomfort of the mice during the experiments. The mice were mated, bred, and genotyped in the animal facility of Case Western Reserve University. All mice used in this study were maintained on a C57BL/6J (Strain #000664, The Jackson Laboratory) background. 5XFAD transgenic mice [Tg(APPSwFlLon,PSEN1*M146L*L286V)6799Vas, strain #034840-JAX] breeders were purchased from Jackson Laboratory.<br><br>The ATAD3A heterogeneous knockout-first mice were obtained from the Wellcome Trust Sanger Institute (Colony name: MGPY; Genetic background: C57BL/6NTac; strain # EPD0159_4_A12). A pair of loxP sites were inserted flanking ATAD3A exon 2, and a LacZ–neomycin cassette flanked with FRT was inserted in intron 1, which terminated Atad3a transcription. The knockout-first mice were then bred with Flp recombinase transgenic mice (129S4/SvJaeSor-Gt(ROSA)26Sortm1(FLP1)Dym/J, strain #003946, The Jackson Laboratory) to remove the LacZ–neomycin cassette and obtain the Atad3a-conditional knockout mice ATAD3Aflox/flox (ATAD3Afl/fl), which contain the ATAD3A allele with exon 2 flanked by LoxP sites. ATAD3Afl/fl mice were bred with CMV-Cre mice (B6.C-Tg(CMV-cre)1Cgn/J, strain #006054, The Jackson Laboratory) to generate CMV;ATAD3Afl/+ heterozygous mice. 5XFAD heterozygous mice were crossed to the CMV;ATAD3Afl/+ mice to generate 5XFADhet;CMV;ATAD3Afl/+ mice. Inbred, age-matched, and sex-balanced WT, CMV;ATAD3Afl/+, 5XFADhet;ATAD3A+/+, 5XFADhet;CMV;ATAD3Afl/+ mice were used for further study.<br><br>Inbred, age-matched, and sex-balanced WT, CMV;ATAD3Afl/+, 5XFADhet;ATAD3A+/+, 5XFADhet;CMV;ATAD3Afl/+ mice were used for further study. Ages of mice between 3 to 8 months were used in the experiments, the detailed information was described in the main text and figure legends. Briefly, male and female WT, CMV;ATAD3Afl/+, 5XFADhet;ATAD3A+/+, 5XFADhet;CMV;ATAD3Afl/+ mice randomly perfromed behaviour test and sacrificed for biochemical analyses at age of 3 months or 8 months.<br><br>5XFAD mice and age-matched and sex-balanced WT littermates were subcutaneously treated with control peptide TAT or DA1 peptide (1mg/kg/day) using Alzet osmotic pump implantation from the age of 1.5 to 9 months. Mice were used at multiple age point from 1.5 to 9 months, the detailed information was provided in the main text and figure legends. Mice were randomly sacrificed for biochemical analyses at age of 6 months or 9 months.<br><br>Both male and female mice were used through all the study, mix-sex analysis was used in all the experiments.
Wild animals	The study did not involve wild animals.
Field-collected samples	The study did not involve field-collected samples.
Ethics oversight	All animal experiments in this study were conducted with protocols approved by the Institutional Animal Care and Use Committee

(IACUC) of Case Western Reserve University and performed according to the National Institutes of Health Guide for the Care and Use of Laboratory Animals. All mice were maintained under a 12 h/12 h light/dark cycle (light on at 6 AM and off at 6 PM) with ad libitum access to food and water under the ambient temperature at 23°C and humidity at 40-60%. Sufficient procedures were employed to reduce the pain and discomfort of the mice during the experiments.

Note that full information on the approval of the study protocol must also be provided in the manuscript.

Human research participants

Policy information about studies involving human research participants

Population characteristics	All postmortem brain samples were collected by the National Institutes of Health (NIH) NeuroBioBank (NBB; https://neurobiobank.nih.gov/) under the approval of the Institutional Review Boards (IRB) and the institution's Research Ethics Board. All brain specimens donated to the NIH NBB were assessed and reviewed by board-certified neuropathologists. A standard assessment was performed to document possible neuropathologies and establish a disease condition diagnosis. In addition, postmortem blood was sampled and submitted for serology and toxicology testing. The human postmortem brain samples used in the experiments were obtained from the NeuroBioBank under a material transfer agreement (MTA) between the NIH and Case Western Reserve University. The detailed information for the human postmortem brains is listed in Supplementary Fig. 2b.
Recruitment	No donor was recruited. All human postmortem brain samples were obtained from the National Institutes of Health (NIH) NeuroBioBank under a material transfer agreement (MTA) between the NIH and Case Western Reserve University.
Ethics oversight	All postmortem brain samples were collected by the National Institutes of Health (NIH) NeuroBioBank (NBB; https://neurobiobank.nih.gov/) under the approval of the Institutional Review Boards (IRB) and the institution's Research Ethics Board. All brains were donated to the NBB through the Brain and Tissue Repositories (BTR) sites. Donation was voluntary and had no financial benefits. All donors provided informed consent and signed a document detailing the goals of the NBB and BTR, participants involvement (questionnaires and tissue sampling), advantages vs risks, compensation, confidentiality measures, rights as participant and contact information.The human postmortem brain samples used in the experiments were obtained from the NeuroBioBank under a material transfer agreement (MTA) between the NIH and Case Western Reserve University. The experiments were performed with the approval of the IRB of Case Western Reserve University.

Note that full information on the approval of the study protocol must also be provided in the manuscript.
